# Supplementary material for: The SARS-CoV-2 main protease doesn’t induce cell death in human cells in vitro
Source: PLoS One. 2022 May 24;17(5):e0266015. doi: 10.1371/journal.pone.0266015 (PMC9129031; doi:10.1371/journal.pone.0266015)
Supplement: S1 Fig — HEK293, HeLa, A549 and Calu1 cells were transfected with p3CL and pm3CL plasmids, and total RNA was isolated 24 h post transfection. The expression of 3CLpro, m3CLpro and ubiquitin C (UBC) genes was detected using quantitative real-time PCR with reverse transcription, and specificity of primer/probe sets was confirmed using corresponding melting curves. (PDF) [file pone.0266015.s001.pdf]

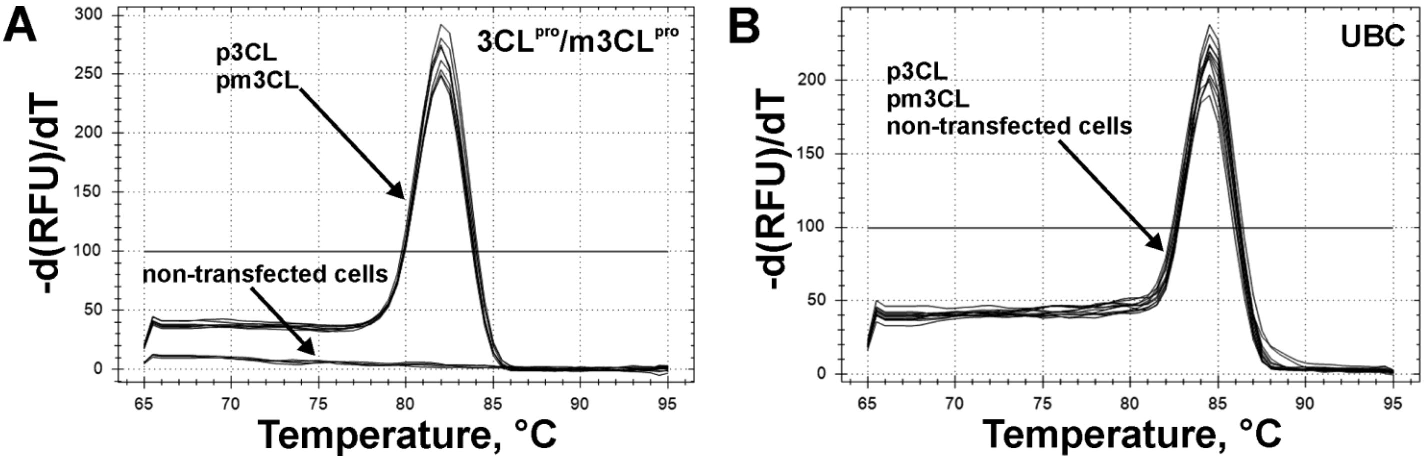

S1 Fig. Specificity of primer/probe sets used for PCR analysis of the expression of 3CLpro/m3CLpro (A) and UBC (B). HEK293, HeLa, A549 and Calu1 cells were transfected with p3CL and pm3CL plasmids, and total RNA was isolated 24 h post transfection. The expression of 3CLpro, m3CLpro and ubiquitin C (UBC) genes was detected using quantitative real-time PCR with reverse transcription, and specificity of primer/probe sets was confirmed using corresponding melting curves.
