# Supplementary material for: The SARS-CoV-2 main protease doesn’t induce cell death in human cells in vitro
Source: PLoS One. 2022 May 24;17(5):e0266015. doi: 10.1371/journal.pone.0266015 (PMC9129031; doi:10.1371/journal.pone.0266015)
Supplement: S2 Fig — Cells were co-transfected with p3CL, pm3CL, or pCI constructs and pGlo-3CL providing the expression of 3CLpro-specific biosensor; 24 h post transfection luciferase activity in transfected cultures was analyzed using GloSensor reagent. Values are represented as mean ± SD of two independent experiments with triplicates (n = 6). (PDF) [file pone.0266015.s002.pdf]

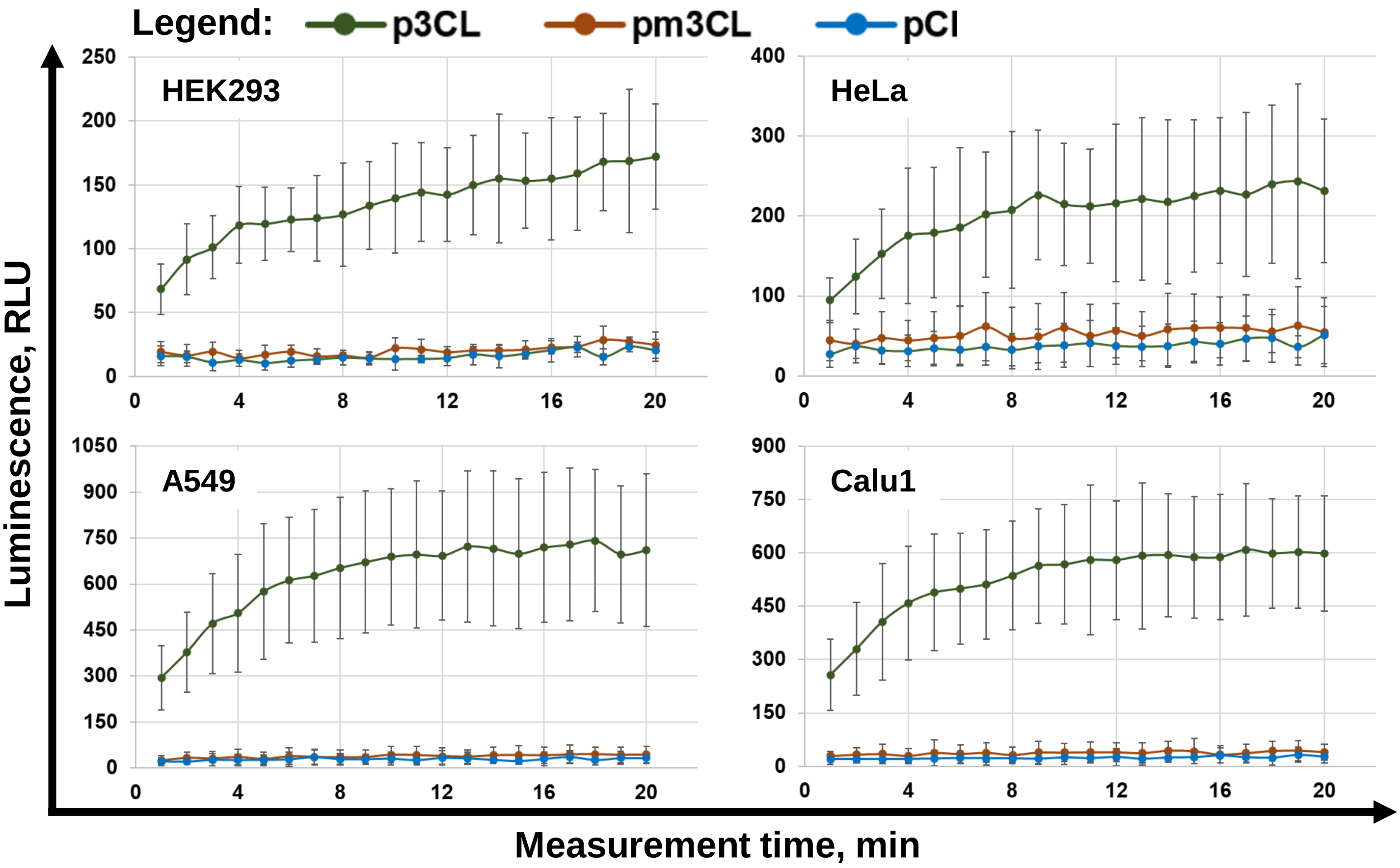

S2 Fig. 3CL^{pro} proteolytic activity analysis. Cells were co-transfected with p3CL, pm3CL, or pCI constructs and pGlo-3CL providing the expression of 3CL^{pro}-specific biosensor; 24 h post transfection luciferase activity in transfected cultures was analyzed using GloSensor reagent. Values are represented as mean ± SD of two independent experiments with triplicates (n = 6).
