# Supplementary material for: The SARS-CoV-2 main protease doesn’t induce cell death in human cells in vitro
Source: PLoS One. 2022 May 24;17(5):e0266015. doi: 10.1371/journal.pone.0266015 (PMC9129031; doi:10.1371/journal.pone.0266015)
Supplement: S1 Table — Values are represented as mean ± SD of two independent experiments with triplicates. No statistically significant differences between the cells expressing 3CL and m3CL were observed (nonparametric two-tailed Mann-Whitney U test, n = 6). h p.t.–hours post transfection. (PDF) [file pone.0266015.s003.pdf]

S1 Table. Characteristics of the cell cultures co-transfected with pCI-EGFP and p3CL/pm3CL plasmids.

Cell line	24 h p.t.		48 h p.t.	
	3CL	m3CL	3CL	m3CL
**% of EGFP-positive cells**				
HEK293	34.2 ± 22.3	31.7 ± 13.5	61.4 ± 14.3	58.1 ± 8.6
HeLa	13.7 ± 2.5	15.3 ± 3.4	37.9 ± 21.4	39.6 ± 9.1
A549	14.5 ± 5.6	15.2 ± 6.8	16.8 ± 7.6	20.6 ± 13.5
Calu1	13.3 ± 7.5	10.8 ± 2.9	16.9 ± 4.2	23.1 ± 11.1
**Mean fluorescence of EGFP-positive cells, RFU**				
**Cell line**	**24 h p.t.**		**48 h p.t.**	
	**3CL**	**m3CL**	**3CL**	**m3CL**
HEK293	869 468 ± 513 692	876 392 ± 485 357	1 193 423 ± 328 292	1 057 723 ± 164 701
HeLa	1 067 964 ± 238 344	976 792 ± 221 189	1 326 672 ± 247 548	1 303 416 ± 404 400
A549	898 868 ± 67 090	895 375 ± 162 922	786 842 ± 307 964	652 890 ± 330 495
Calu1	850 300 ± 159 658	792 967 ± 149 797	801 678 ± 498 137	758 177 ± 466 184

Values are represented as mean ± SD of two independent experiments with triplicates. No statistically significant differences between the cells expressing 3CL and m3CL were observed (nonparametric two-tailed Mann-Whitney U test, n =6). h p.t. – hours post transfection.
