# Supplementary material for: The SARS-CoV-2 main protease doesn’t induce cell death in human cells in vitro
Source: PLoS One. 2022 May 24;17(5):e0266015. doi: 10.1371/journal.pone.0266015 (PMC9129031; doi:10.1371/journal.pone.0266015)
Supplement: Attachment — Submitted filename: response to reviewers.docx [file pone.0266015.s004.docx]

First of all, together with co-authors we would like to thank the referees for thorough examination of our work. The referees provided us with valuable remarks which we tried to answer in the current revised manuscript. Point-to-point response to the reviewers’ comments is presented below.

**Response to the Reviewer #1 comments:**

*– “The paper is reasonably well written, although there are several English language grammatical errors, especially involving comma usage.”*

The manuscript text was edited by a native English speaker who is a professional in the subject area of the paper (throughout the text).

*– “The most significant weakness of this submission is that no evidence is provided that the SARS-CoV-2 wildtype or catalytically inactive 3CL proteases are actually produced in the transiently transfected cells, and the entire study rests upon this. RT-qPCR data is presented, which shows that RNA transcripts are generated in the transfected cells after 24 hours, but mRNA synthesis is only one of many steps of gene expression that result in the appearance of a protein, at a particular concentration and at a particular time. It is crucial that the authors demonstrate that transfection with their expression vectors leads to the generation of the 3CL proteins within the timeframe of the subsequent cell viability experiments. This could be accomplished by Western blotting or immunofluorescence. Do their vectors produce transcripts that can be translated? Some 3C and 3CL proteases (including the HAV 3C protease, used as a control in this study) are subject to rapid degradation by the ubiquitin-proteasome system, and no information exists concerning the stability of the SARS-CoV-2 3CLpro or, for that matter, the SARS-CoV 3CLpro. It should be noted that in the SARS-CoV 3CLpro apoptosis study cited by the authors (ref. 11, Lin etal. 2006), the presence of the 3CLpro in stably transfected cells was proven, although the kinetics of 3CLpro production and its stability were not, a fact which somewhat lessens the significance of the cited study. It is possible that the authors of the current study did not observe cytotoxicity due to SARS-CoV-2 CL3pro because little or no 3CLpro proteins were present in the cells.”*

We agree that showing only mRNA expression when the effects of a proteolytic enzyme are studying is not enough, as well as protein production shown by Western blot since it provides no information about the activity. So, in the revised manuscript we analyzed the proteolytic activity of the 3CLpro. We created a luciferase-based biosensor similar to the one described for 3C-like protease of the Middle East respiratory syndrome coronavirus. This biosensor is based on the GloSensor technology developed by Promega and represents inactive circular permutated firefly luciferase. The wild-type N- and C-termini of the enzyme are linked by a polypeptide containing the 3CLpro recognition site (AVLQ↓S) corresponding to the SARS-CoV-2 polyprotein nsp4/5 junction. A conformational change in the biosensor upon cleavage by 3CLpro restores the luciferase catalytic activity. We showed that robust luciferase signal was observed in cells co-expressing the biosensor and 3CLpro, while no signal was detected in cells both expressing inactive m3CLpro and transfected with intact pCI vector. The results of the experiment are shown in Figure 1B and Supplementary Figure S2 and are discussed in the text (Page 9, Lines 194-207).

*– “The data presented in Figure 2 suggest that the HAV 3C protease is generated from the expression vector, since a reduction in cell viability was observed, but the authors do not present data for cells concurrently transfected with the catalytically inactive HAV 3Cpro-encoding plasmid they prepared, p3Cmut, to demonstrate that this effect in their system and in these experiments is actually due to HAV 3C protease activity.”*

These plasmids were used just as controls in order to prove that the experimental system used was working. The fact that cytotoxic activity of HAV 3Cpro depends on the proteolytic activity of the enzyme has been shown previously in our laboratory (Shubin et al. Biologia. 2021, 76:321-7; Komissarov et al. Mol Biol Rep. 2017, 44:323-32; Shubin et al. BMC Cell Biol. 2015, 16:4; Shubin et al. Appl Biochem Microbiol. 2013, 49:750-5). Thus, we decided not to include this information in the main text because of its redundancy. However, in accordance with the Reviewer comments we added these results in Figure 2.

*- “This study would be strengthened, whether reagent-based cell viability or mitochondrial dye-based flow cytometry indicate 3CL-induced cytotoxicity or not, by some additional evidence for or against apoptosis. For example, fluorometric caspase activity measurements could be conducted”.*

The aim of the current study was to analyze the cytotoxic effects of 3CLpro expression. Based on the previous data obtained for SARS-CoV 3CLpro we assumed that the cytotoxicity of the SARS-CoV-2 3CLpro is likely to result in apoptosis induction. However, it still was possible that the enzyme either induced different type of regulated cell death or possessed no cytotoxicity. In this context, we needed to confirm that cells basically die upon the enzyme expression before we could pass to the analysis of the cell death type. For this purpose, we started our analysis from cell death markers that are common for the most of cell death types and are considered as points-of-no-return, since regulated cell death is a reversible process. Thus, we studied mitochondrial activity and cytoplasmic membrane integrity and found that the enzyme expression didn’t result in cell death. So, we believe that subsequent experiments aimed to detect specific features of different cell death types, including caspase activation, are impractical. However, the Reviewer’s comment forced us to reconsider the title of the manuscript. Thus, we decided to substitute “apoptosis” for “cell death”.

*- “Assuming their vectors do support the verifiable production of 3CLpro proteins, perhaps the authors should consider examining the long-term stability of both the SARS-CoV and SARS-CoV-2 3CL proteins. This could be accomplished, for example, by pulse-chase measurements using 35S. Proteasome inhibitors could be used in a preliminary evaluation of whether or not these proteins are ubiquitin-proteasome system substrates. This would be important information to have. It would also be helpful to know, for the sake of confirming biological relevance, whether the 3CL levels in the transiently transfected cells approximate those that appear in SARS-CoV-2-infected cells.”*

In the current revised version of the paper we showed that the vectors constructed provided for the expression of the catalytically active enzyme. However, we also showed that SARS-CoV-2 3CLpro did not induce cell death in contrast to homologous proteases. It is not clear what features underlie this difference in cytotoxicity between the enzymes. To clarify this issue, it is required to perform additional experiments, which surely should include but not limited to the ones suggested by the Reviewer, and it is surely worth independent research. However, this question is beyond the scope of the current study aimed to analyze the presence of cytotoxic effects of the 3CLpro.

**Response to the Reviewer #2 comments:**

*- “The experiments are well done however the simple control is lacking to confirm proteolytic activity of the 3CLmain expressed in the target cells. It is well known that dimerization is required for 3Cl activity. Without a control for this then the conclusions are invalid. Thus, a dual transfection of the 3Clmain with the sequence of two of the viral proteins that are cleaved by the enzyme and a suitable reporter system or assay developed to confirm cleavage. Only then can the induction or not of apoptosis in the transfected cells be confirmed.”*

According to the Reviewer’s comments in the revised manuscript we analyzed the proteolytic activity of 3CLpro using a luciferase-based biosensor which we created for this purpose. This biosensor is similar to the one described for 3C-like protease of the Middle East respiratory syndrome coronavirus. The biosensor is based on the GloSensor technology developed by Promega and represents inactive circular permutated firefly luciferase. The wild-type N- and C-termini of the enzyme are linked by a polypeptide containing the 3CLpro recognition site (AVLQ↓S) corresponding to the SARS-CoV-2 polyprotein nsp4/5 junction. A conformational change in the biosensor upon cleavage by 3CLpro restores the luciferase catalytic activity. We showed that robust luciferase signal was observed in cells co-expressing the biosensor and 3CLpro, while no signal was detected in cells both expressing inactive m3CLpro and transfected with intact pCI vector. The results of the experiment are shown in Figure 1B and Supplementary Figure S2 and are discussed in the text (Page 9, Lines 194-207).
